# Relationship Between Nasal Cycle, Nasal Symptoms and Nasal Cytology

**DOI:** 10.1177/1945892419858582

**Published:** 2019-06-20

**Authors:** Alfonso Luca Pendolino, Bruno Scarpa, Giancarlo Ottaviano

**Affiliations:** 1 Department of Neurosciences, Otolaryngology Section, University of Padova, Padova, Italy; 2 Department of Statistical Sciences, University of Padova, Padova, Italy

**Keywords:** nasal cycle, nasal cycle patterns, nasal mucosa, nasal cytology, rhinitis, local nasal inflammation, peak nasal inspiratory flow, Sino-Nasal Outcome Test-22 questionnaire, Visual Analog Scale

## Abstract

**Background:**

The nasal cycle is the spontaneous congestion and decongestion of nasal mucosa that happens during the day. Classically, 4 types of nasal cycle patterns have been described: (1) classic, (2) parallel, (3) irregular, and (4) acyclic. Hypothalamus has been considered as the central regulator even if several external factors may influence its activity.

**Objective:**

The aim of the study was to evaluate the presence of a correlation between nasal cycle pattern, nasal cytology and nasal symptoms.

**Methods:**

Thirty healthy volunteers have been enrolled in the study. All subjects completed a Sino-Nasal Outcome Test-22 questionnaire and a Visual Analog Scale (VAS) for nasal obstruction. The nasal cycle was studied by means of peak nasal inspiratory flow. Nasal cytology has been used to evaluate the presence of local nasal inflammation.

**Results:**

Nineteen subjects showed a parallel nasal cycle pattern, while 11 showed a regular one. A parallel pattern was present in 60% of asymptomatic subjects and in 67% of the symptomatic one (*P* = 1). VAS for nasal obstruction did not show a significant difference between the 2 patterns of the nasal cycle (*P* = .398). Seventeen subjects had a normal rhinocytogram, while 13 volunteers showed a neutrophilic rhinitis; 53.8% of the subjects with a neutrophilic rhinitis showed a parallel pattern, while the remaining 46.2% had a regular one. In the case of a normal cytology, 70.6% of the volunteers had a parallel pattern and 29.4% had a regular one. Differences between the 2 groups were not statistically significant (*P* = .575).

**Conclusion:**

Rhinitis with neutrophils seems to not influence the nasal cycle pattern. Based on the present results, the pattern of nasal cycle does not influence subjective nasal obstruction sensation.

## Introduction

The nasal cycle (NC) is the spontaneous congestion and decongestion of the nasal mucosa during the day, where congestion of one side is generally accompanied by reciprocal decongestion of the contralateral side. It is accepted that almost 70% to 80% of adults experience a regular NC, but a true periodicity/reciprocity exists only in 21% to 39% of the population.^1–3^ Classically, 4 types of NC’s patterns have been described with frequencies reported for each pattern often discordant. These include (1) classic (reciprocal congestion/decongestion alterations and a constant total volume), (2) parallel (congestion or decongestion appearing in both nasal cavities at the same time), (3) irregular (mutual alteration in nasal volume without a defined pattern and a constant total nasal volume), and (4) acyclic (total nasal volume and nasal volume in each nostril do not differ).^4^

Congestion and decongestion of the nasal venous cavernous tissue is under the control of the autonomous nervous system,^5–7^ even if the central regulation of the sympathetic activity at the level of the nose is not completely known. Recently, Williams and Eccles proposed a control model involving a hypothalamic center and 2 brainstem half centers.^8^ However, several conditions may influence this central regulation. In particular, the presence of an infectious or an allergic rhinitis has been showed to interfere with the spontaneous congestion and decongestion in the context of the NC by leading to a modification in its amplitude and frequency.^9–12^

According to recent data, it is estimated that more than 200 million people worldwide suffer from nonallergic rhinitis (NAR).^13,14^ The diagnosis of the specific type of rhinitis can be something challenging. Nasal cytology has been shown to be a useful and easy diagnostic tool in the study of rhinitis,^15,16^ as it allows to detect and measure the cell population within the nasal mucosa at a given instant, to better discriminate different pathological conditions and also to evaluate the effects of various stimuli (allergens, infections, irritants, physical activity^17^).

The aim of this study was to evaluate if the presence of a local nasal inflammation evaluated by means of nasal cytology could influence the type of NC pattern. As a second outcome, we wanted to investigate if the type of nasal pattern may influence nasal obstruction sensation.

## Materials and Methods

A cohort of 30 healthy adult volunteers ranging from 23 to 42 years, with a mean age of 29 ± 4.7 years, was recruited at the Department of Neurosciences, Section of Otolaryngology of Padova University. All subjects were asked to complete only at the beginning of the day a Sino-Nasal Outcome Test (SNOT)-22 questionnaire and a Visual Analog Scale (VAS) for the symptom “nasal obstruction.” Weight and height were also collected. Volunteers were also asked if they were smokers, asthmatic, or had undergone any previous surgery on the nose and paranasal sinuses. All the subjects who were nonsmokers, nonasthmatic, and without any previous sinonasal surgery were enrolled in the study. Subjects with an infectious rhinitis or an allergic rhinitis during the active phase of pollen exposure were also excluded. None of the subjects enrolled took any form of medication. Detailed characteristics of the population are reported in Table 1. The present investigation was conducted in accordance with the 1996 Helsinki Declaration. Written informed consent was obtained from each subject before starting any study-related procedure. Data were examined in agreement with the Italian privacy and sensible data laws (D.Lgs 196/03) and the internal regulation of the sections involved.

**Table 1.** Detailed Characteristics of the Population.

	Asymptomatic (n = 15)			Symptomatic (n = 15)		
Variables	Mean Value	Standard Deviation	Range	Mean Value	Standard Deviation	Range
Age (years)	28	2.8	24–34	30	5.1	23–42
Height (cm)	170.5	7.1	158–180	171.4	8.9	153–187
Weight (kg)	63.7	11.5	47–81	68.7	17.7	50–115
BMI (kg/m^2^)	21.7	2.5	18.3–25.2	23.1	3.9	18.4–32.9
lPNIF (L/min)	82.7	34.7	35–130	86.7	45.3	30–185
rPNIF (L/min)	89	35.9	45–160	86	28.5	30–140
PNIF (L/min)	152.3	56.4	70–265	145	48.5	65–265
SNOT-22	8.5	4.5	2–18	30.5	8.6	22–48
VAS (nasal obstruction)	1.5	2.1	0–6	4.3	2.6	0–8

Abbreviations: BMI, body mass index; PNIF, peak nasal inspiratory flow; SNOT-22, Sino-Nasal Outcome Test-22; VAS, Visual Analog Scale.

Based on the score obtained at SNOT-22,^18^ all the volunteers were divided into 2 groups: the first group comprised 15 subjects (7 males and 8 females) with moderate to severe nasal symptoms (SNOT-22 ≥ 22) and the second one comprised 15 subjects (7 males and 8 females) with mild nasal symptoms (SNOT-22 < 22). NC was studied by means of peak nasal inspiratory flow (PNIF), as previously done.^19^ A portable Youlten peak flow meter (Clement Clark International) was used for the PNIF measurement. Unilateral PNIF (lPNIF and rPNIF) was also measured as previously reported.^20^ All nasal measurements were obtained 4 times in a single day, at 08.30, 11.00, 13.30 and 16.00. For PNIF and unilateral PNIF, 2 satisfactory maximal inspirations were obtained each time, and the higher of the 2 results was then considered. All PNIF measurements were performed in all participants after at least 10 minutes of acclimatization in a room with constant temperature (between 19°C and 22°C) and a relative humidity of 25% to 35%,^2^ by the same operator (A. L. P.).

Nasal cytology was performed at 8.30 as the first exam. Nasal mucosal samples were obtained by collecting nasal mucus from the middle portion of the inferior turbinate with a curette under anterior rhinoscopy and an appropriate light source. The sample was then immediately smeared on a glass slide and air-dried. Then, the slide was stained with the common May–Grunwald–Giemsa procedure, and the stained sample was read at optical microscopy with a 100× objective with oil immersion. At least 5 fields were read to obtain a mean value of the differential cellular count.^21^ Nasal cytology analysis was performed by the same operator (G. O.).

### Statistical Analysis

Pearson correlation test was used to compare PNIF, lPNIF, and rPNIF in the evaluation of nasal airflow variations. *P* values have been calculated for all tests, and 5% was considered as the critical level of significance. The pattern of nasal airflow for each subject was expressed as a Pearson’s correlation coefficient, where a positive value indicates a direct correlation of left and right airflows with the changes in parallel, and a negative correlation coefficient indicates a reciprocal correlation of left and right nasal airflows. χ^2^ test with Yates’ correction has been used to measure connection between nasal airflows classification, symptomatology, and type of cytology. Multiple logistic regression with selection of variable based on Akaike’s information criterion (backward stepwise) has also been performed to identify connections between the available variables and the type of nasal airflow. The R: a language and environment for statistical computing (R Foundation for Statistical Computing, Vienna, Austria) was used for all analyses.

## Results

**Figure 1.**
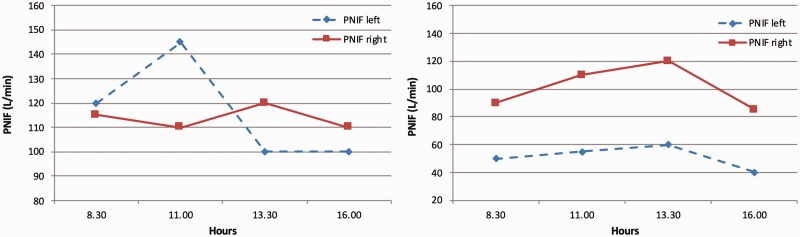
Example of a parallel (*left*) and classic (*right*) pattern of nasal cycle evaluated by means of PNIF in a period of 7.5 hours in 2 of the subjects enrolled. PNIF, peak nasal inspiratory flow.

Figure 1 shows NC evaluated by means of PNIF in a period of 7.5 hours in 2 of the subjects enrolled. Table 1 reports mean values, standard deviations, and ranges for all the variables studied in the population.

**Figure 2.**
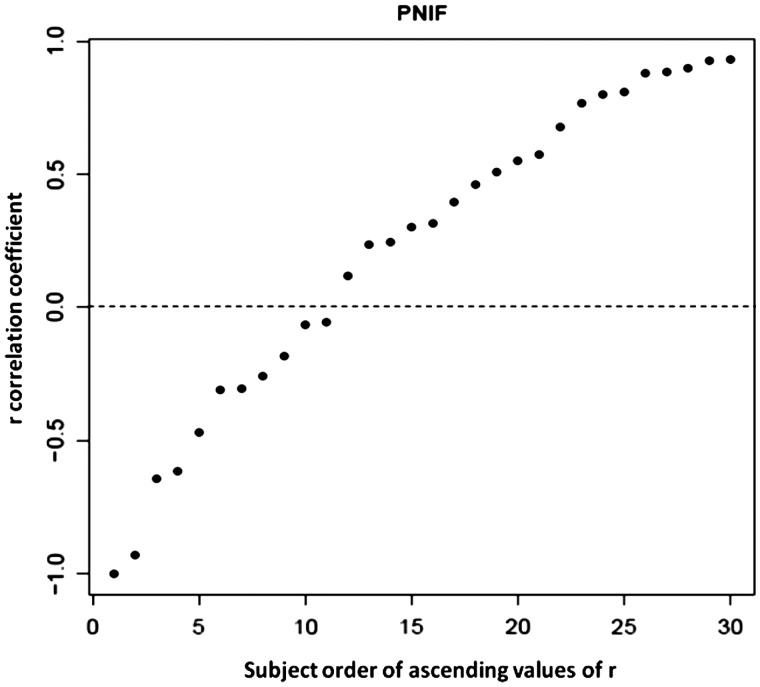
Correlation coefficient *r* of PNIF measurements, describing the relationship between the changes in nasal airflows on each side of the nose. An *r* < 0 means a classic pattern of the nasal cycle, while an *r* > 0 means a parallel pattern of nasal cycle. PNIF, peak nasal inspiratory flow.

**Figure 3.**
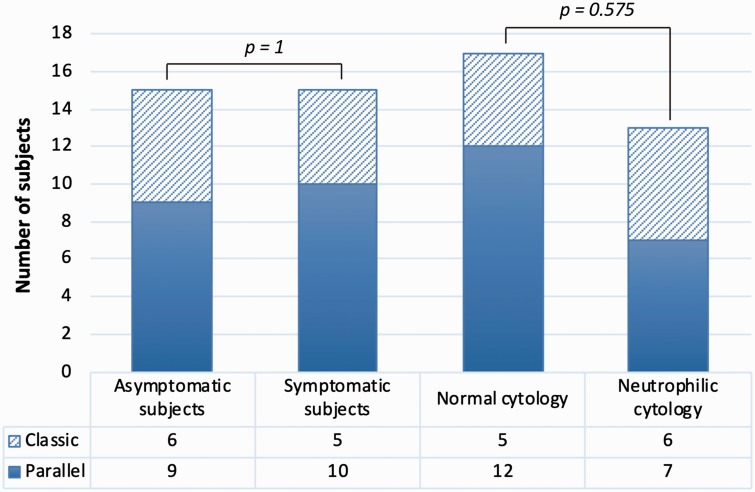
Representation of nasal cycle patterns according to the different groups.

Considering all the 30 subjects, 19 (63.3% of the population) presented a parallel pattern of NC and 11 (36.7% of the population) showed a classic one (Figure 2). Based on the score obtained at the SNOT-22, a parallel pattern was found in 60% (9/15) of the asymptomatic volunteers and in 67% (10/15) of the symptomatic ones. Considering the type of pattern, no significant difference between the 2 groups was found (*P* = 1) (Figure 3). Also considering only the symptom “nasal obstruction,” measured by means of VAS, we did not observe a statistically significant difference between the 2 patterns (*P* = .398).

Nasal cytology revealed a neutrophilic rhinitis in 13 volunteers, while the remaining 17 subjects showed a normal ratio of the various cell types. By evaluating the distribution of the 2 NC patterns according to the nasal cytology diagnosis, we observed that 53.8% (7/13) of the subjects with a neutrophilic rhinitis had a parallel pattern of NC, while the remaining 46.2% (6/13) had a classic pattern. Moreover, when a normal nasal cytology was observed, 70.6% (12/17) of the volunteers showed a parallel pattern of NC, while the remaining 29.4% (5/17) showed a classic one (Figure 3). However, no significant difference was observed in the distribution of the 2 patterns according to nasal cytology (*P* = .575). In addition, in a multiple logistic regression analysis, also considering the effect of the other variables available (sex, age, weight, height, and history positive for allergy), nasal cytology did not show a significant difference in relation to NC pattern.

## Discussion

NC is a complex phenomenon and the mechanism which regulates nasal mucosa sympathetic activity alternation is still not completely known. Hypothalamus is believed to play as the central regulator of this cyclical activity, as it has been observed that its electrical stimulation in cats evokes bilateral nasal vasoconstrictor responses.^22^ In addition, no NC can be revealed in patients with Kallman syndrome.^23^

Classic pattern is generally considered to be the most common in general population.^4,8^ However, in our study, the parallel pattern was more frequent (63.3% of the subjects) than the classic one. A similar result has been already reported in a previous study conducted on 20 healthy volunteers.^19^ To the best of our knowledge, no previous studies have investigated the relationship between NC pattern and nasal obstruction sensation or other nasal symptoms. It could be argued that subjects with a parallel pattern of NC can experience higher fluctuations of nasal airflows during the day than those with a classic NC pattern. In the latter, in fact, the reciprocal congestion/decongestion of the 2 sides is generally associated with a constant total nasal airflow. In our study, we could not find a difference in the distribution of NC pattern in relation to the referred nasal symptoms (*P* = 1). Parallel pattern was indeed the most common pattern both in asymptomatic subjects and in symptomatic ones (60% of the asymptomatic volunteers and 67% of the symptomatic ones), showing that NC pattern was not correlated with the nasal symptoms reported by the volunteers. In addition, also considering the VAS for nasal obstruction, we did not find a significant difference between the 2 patterns (*P* = .398). Also, this result suggests that having a specific type of pattern (classic or parallel) is not responsible for a worse nasal obstruction sensation.

Data in the literature report that NC can be demonstrated in 70% to 80% of adults, even if the majority of them are not conscious to experience an NC, but tend to notice it occasionally, especially during nasal inflammatory diseases. Rhinitis, both infectious and allergic, has been shown to interfere with NC expression. The inflammation of the nasal mucosa, in fact, causes the vasodilatation of the resistance vessels and then an increased filling pressure of the nasal sinusoids, with consequent nasal congestion.^2,24^ In 1989, Bende et al. observed an increase in the NC amplitude after the inoculation of nasal drops containing a Coronavirus.^24^ In a similar way, Eccles found that the amplitude of the spontaneous reciprocal changes in nasal airway resistances increases during acute upper respiratory tract infection due to the increased level of unilateral nasal congestion.^25^ Considering allergic rhinitis, Huang et al. observed greater amplitudes of nasal patency fluctuation in subjects with perennial allergic rhinitis when compared to healthy subjects.^26^ Nasal challenge test generally increases the amplitude of the NC in allergic rhinitis patients; however, it does not alter the occurrence and the period of the NC, which remains under the control of the central nervous system.^11,12,27^

According to recent estimates, about 200 million people worldwide suffer from non-infectious-non-allergic rhinitis (ie, NAR), and its prevalence is still increasing.^13^ Nasal cytology has been shown to be a useful tool in the diagnosis of rhinitis and in particular in the diagnosis of NAR.^15,16^ In the present population, composed of 30 healthy subjects, 17 of them had a normal nasal cytology, while the remaining 13 had a neutrophilic nasal cytology. We did not find neither eosinophils nor mastocytes in the nasal cytology of the volunteers enrolled probably because subjects with allergic rhinitis in an active phase, asthma, or nasal polyps were excluded from the study.^28^ Therefore, the subjects of our population with a neutrophilic nasal cytology could have had an NAR or an allergic rhinitis with a low dose of allergen exposure (eg, house dust mite).^28^ Interestingly, we did not observe a significant difference in NC pattern’s expression between subjects with neutrophilic (53.8% parallel pattern and 46.2% classic pattern) or normal nasal cytology (70.6% parallel pattern and 29.4% classic pattern) (*P* = .575). Furthermore, parallel pattern of NC was the most frequent both in the whole population (63.3% of the volunteers) and in the nasal cytology subgroups (70.6% of the subjects with a normal nasal cytological study and 53.9% of those with neutrophils at the nasal cytology). These results suggest that the presence of a nasal mucosa neutrophilic inflammation does not influence the NC pattern. In this regard, in the near future, it would be interesting to perform a nonspecific nasal provocation test by means of cold air^29^ or hyperosmolar solutions^30^ in subjects with neutrophils in the nasal smear in order to better evaluate if their presence/number could influence the NC.

## Conclusions

Several conditions acting at the level of the nasal mucosa can influence NC expression. The present investigation is the first that has evaluated if there is a correlation between nasal inflammation and NC pattern. According to our findings, the presence of a neutrophilic rhinitis does not influence the pattern of NC, which would remain under the control of the central nervous system. In addition, the present results suggest that the presence of a specific pattern of NC is not accountable for a worse nasal obstruction sensation. Finally, once more, the parallel pattern of NC has been shown to be the most common. Further studies based on larger series and in a multicentric setting are needed to confirm these interesting results, especially in patients affected by neutrophilic rhinitis.
